# Perspective: Extending the Utility of Three-Dimensional Organoids by Tissue Clearing Technologies

**DOI:** 10.3389/fcell.2021.679226

**Published:** 2021-06-14

**Authors:** Etsuo A. Susaki, Minoru Takasato

**Affiliations:** ^1^Department of Biochemistry and Systems Biomedicine, Graduate School of Medicine, Juntendo University, Tokyo, Japan; ^2^Laboratory for Synthetic Biology, RIKEN Center for Biosystems Dynamics Research, Osaka, Japan; ^3^Laboratory for Human Organogenesis, RIKEN Center for Biosystems Dynamics Research, Kobe, Japan; ^4^Laboratory of Molecular Cell Biology and Development, Department of Animal Development and Physiology, Graduate School of Biostudies, Kyoto University, Kyoto, Japan

**Keywords:** organoid, tissue clearing technique, 3D imaging, light-sheet fluorescence microscopy, omics

## Abstract

An organoid, a self-organizing organ-like tissue developed from stem cells, can exhibit a miniaturized three-dimensional (3D) structure and part of the physiological functions of the original organ. Due to the reproducibility of tissue complexity and ease of handling, organoids have replaced real organs and animals for a variety of uses, such as investigations of the mechanisms of organogenesis and disease onset, and screening of drug effects and/or toxicity. The recent advent of tissue clearing and 3D imaging techniques have great potential contributions to organoid studies by allowing the collection and analysis of 3D images of whole organoids with a reasonable throughput and thus can expand the means of examining the 3D architecture, cellular components, and variability among organoids. Genetic and histological cell-labeling methods, together with organoid clearing, also allow visualization of critical structures and cellular components within organoids. The collected 3D data may enable image analysis to quantitatively assess structures within organoids and sensitively/effectively detect abnormalities caused by perturbations. These capabilities of tissue/organoid clearing and 3D imaging techniques not only extend the utility of organoids in basic biology but can also be applied for quality control of clinical organoid production and large-scale drug screening.

## Introduction

Directed differentiation of human pluripotent stem cells (PSCs) to generate target organ cells is currently the most promising method to create artificial organs for regenerative medicine. To date, various cell types of target organs have been induced, including those of the blood, myocardium, lung, pancreas, liver, intestine, brain, and kidney (Wang et al., 2007; Davis et al., 2008; Yang et al., 2008; Xia and Zhang, 2009; Spence et al., 2011; Pagliuca et al., 2014; Takasato et al., 2014). However, reproducing the original function of organs with the use of differentiated cells remains a significant challenge, as correct replication of the three-dimensional (3D) structures of the original organ is essential.

A method to create 3D mini-organs from human PSCs has continued to attract attention in recent years. When organ-specific progenitor cells derived from human PSCs are aggregated and cultured under 3D culture conditions, the progenitor cells undergo self-organization within the aggregate to form organ-specific tissues in the same manner that occurs during ontogenesis. Such aggregates are called *organoids*. Ocular organoids were the first to be generated, which was followed by the creation of gastric, liver, brain, intestinal, and renal organoids (Eiraku et al., 2011; Lancaster et al., 2013; Takebe et al., 2013; McCracken et al., 2014; Takasato et al., 2015). Organoids can potentially contribute to various fields of research, including tissue physiology, stem cell biology, developmental biology, disease modeling, drug discovery, and regenerative medicine (Foglietta et al., 2020; Grenier et al., 2020; Li et al., 2020; Bock et al., 2021).

Due to the intrinsic 3D organ-like architecture of organoids with random coordinates in contrast to the corresponding native tissues, 3D observation is essential to obtain accurate structural information of dynamic 3D events (Rios and Clevers, 2018). However, the intense scattering of light within organoids limits 3D observation to the sub-millimeter range from the surface. In addition, 3D reconstitution by serial sectioning is arduous due to the fragility of organoids, which leads to deformation and fracturing of the sample, resulting in insufficient resolution, low contrast, and lack of internal 3D characterization (Pampaloni et al., 2013; Renner et al., 2017). A biased sampling of two-dimensional sections may result in inaccurate quantitative data with large standard deviations (Albanese et al., 2020).

Recent tissue clearing and 3D imaging technologies have the potential to solve these problems and provide system-level single-cell analysis of whole multicellular structures. 3D imaging of large tissue samples of whole organs and bodies has already been established by combining efficient tissue clearing protocols and optical microscopy, which provides useful information of the unique 3D structures of biological tissues by collecting images of the entire sample (Susaki and Ueda, 2016; Ueda et al., 2020a,b). In addition, tissue clearing protocols for spheroids and organoids have been developed and even applied in recent studies (Costa et al., 2019). Although each tissue clearing protocol has unique advantages and disadvantages, optimization of relatively small organoid samples is easier than that of large and complicated animal tissues. Besides, detailed 3D analysis of a single organoid can expedite the clearing and 3D imaging framework for a large-scale multi-organoid screening approach to assess genetic or pharmacological perturbations. Therefore, the aim of this perspective is to summarize recent adaptations and prospects of tissue clearing and 3D imaging frameworks in organoid research.

### Brief Overview of Modern Tissue Clearing Technologies

Tissue clearing is basically an extension of the conventional histology technique that literally makes tissue “transparent” by suppressing light scattering and light absorption in the fixed tissue sample. Since Lundvall and Spalteholz initialized clearing of human tissues with the use of organic solvents more than 100 years ago (Lundvall, 1905; Spalteholz, 1914), the field has achieved dramatic innovations in the last few decades, as dozens of protocols, categorized as organic solvent (hydrophobic reagent)-based protocols, hydrophilic reagent-based protocols, and hydrogel-tissue chemistry (Ueda et al., 2020a,b), have been recently established.

Optical clearing generally involves exchanging the surrounding medium (e.g., phosphate-buffered saline) with a solvent with optical properties similar to those of the biomaterial. This step is called refractive index (RI) matching because the RI is a preferred indicator of optical properties. However, this process is relatively complex physicochemically and not necessarily limited to RI homogenization, as dispersion of the fibrous structures of the extracellular matrix and the affinity (infiltration) of compounds into living tissues may also have significant effects (Ueda et al., 2020a; Yu et al., 2021). RI matching can also be combined with other processes to remove light-scattering and light-absorbing substances from the biological tissue, such as lipids (delipidation), the bone matrix (decalcification), and pigments (decolorization or bleaching). The requirement of incorporating these steps is dependent on the experimental purpose and target tissue type. Tissue clearing protocols incorporate these steps by combining various compounds and physical techniques (e.g., electrophoresis).

Organic solvent-based tissue clearing protocols originating from the Spalteholz reagent are represented by the benzyl alcohol/benzyl benzoate (BABB) method, 3D imaging of solvent-cleared organs (3DISCO), and the ethyl-cinnamate (ECi) method (Dent et al., 1989; Becker et al., 2012; Ertürk et al., 2012; Klingberg et al., 2017). Generally, these protocols have very strong clearing abilities over relatively short periods of time. However, it is necessary to understand the characteristics of the reagents regarding tissue shrinkage, signal retention of fluorescent proteins, safety, and compatibility with the microscope system.

The hydrophilic reagent-based clearing technique was initially tested with the use of several hydrophilic chemicals (e.g., sugars and alcohols) to transluce human skin and sclera in medical applications (Bakutkin et al., 1995; Zimnyakov et al., 1996; Tuchin et al., 1997). Since then, a wide variety of hydrophilic reagent protocols have been proposed with the advantages of ease of handling, safety, and preservation capacity of biomaterials, which include FocusClear^TM^ (Chiang et al., 2001), Sca*l*e (Hama et al., 2011, 2015), *Clear*^*T*^ (Kuwajima et al., 2013), SeeDB (Ke et al., 2013, 2016), FRUIT (Hou et al., 2015), CUBIC (clear, unobstructed brain/body imaging cocktail and computational analysis) (Susaki et al., 2014; Tainaka et al., 2018), FUnGI (fructose, urea, and glycerol for imaging) (Rios et al., 2019), RTF (rapid clearing method based on triethanolamine and formamide) (Yu et al., 2018), Ce3D (clearing-enhanced 3D) (Li et al., 2017), and TDE (2,2′-thiodiethanol) immersion (Aoyagi et al., 2015).

Hydrogel-tissue chemistry involves the preparation of a tissue-hydrogel scaffold by cross-linking hydrogel monomers to native biomolecules (Gradinaru et al., 2018). The initial formulation, called CLARITY (and its variations), uses acrylamide, while later versions, SWITCH (Murray et al., 2015) and SHIELD (Park et al., 2018), utilize glutaraldehyde and a polyepoxide, respectively. Due to the increased tissue rigidity caused by transformation, these protocols can be combined with harsh delipidation or re-probing procedures with sodium dodecyl sulfate (SDS) and physical electrophoresis. Furthermore, the use of a water-absorbing polymer for Expansion Microscopy (ExM) enables high-resolution imaging with a general microscopy setup (Chen et al., 2015; Ku et al., 2016). The expansion can also contribute to the final transparency of the sample. Other hydrophilic clearing reagents have also applied the same strategy (Kim et al., 2018; Murakami et al., 2018; Tainaka et al., 2018).

Although limited to fixed samples, tissue clearing facilitates observation of the 3D architecture of biological tissues with a light microscope at the cellular or higher resolution, thereby providing a powerful analytical approach for complex biological systems.

### Tissue Clearing Technologies in Organoid Studies

Clearing of cell reaggregates (spheroids and organoids) for whole-mount imaging was recommended in early protocols (Timmins and Nielsen, 2007). More recently, many of the clearing methods introduced in the previous section have been tested from simple whole-mount observations to advanced phenotyping of 3D cultured reaggregates (Costa et al., 2019; Table 1). Since most current clearing protocols are optimized for animal tissues and organs, clearing cell reaggregates with the use of any of these procedures is, in principle, much simpler. Although tissue clearing methods have been adopted at least to some extent, experience is required for further applications in broader organoid research.

**TABLE 1:** Recent tissue clearing applications in organoid research.

Method category	Protocol	Chemical	Applied reaggregates	References
Organic solvent-based method	BABB	Ethanol Hexane Benzyl benzoate Benzyl alcohol	Cultured cancer cell spheroids Human midbrain organoids	Wenzel et al., 2014 Smyrek and Stelzer, 2017 Schmitz et al., 2017 Desmaison et al., 2018 Messal et al., 2021 Renner et al., 2020
	3DISCO	Tetrahydroflurane Dichloromethane Dibenzylether	Human iPSC-derived retinal organoids	[Bibr B88][Bibr B33]
	ECi method	Ethyl cinnamate	Human cerebral organoids Human brain organoids co-cultured with patient-derived glioblastoma cells Vascularized tumor and neural organoids	Masselink et al., 2019 Goranci-Buzhala et al., 2020 Wörsdörfer et al., 2019
Hydrophilic reagent-based method	Single chemical	Urea Glycerol TDE	Tumor cell spheroids Tumor cell spheroids Various human cell spheroids Pancreatic tumor cell spheroids Tissue spheroids	Wei et al., 2019 Timmins and Nielsen, 2007 Nürnberg et al., 2020 Steinberg et al., 2020 Paiè et al., 2016
	*Clear*^*T*^	Formamide Polyethylene glycol	Rat neural cell and glioma cell spheroids Human cell spheroids Human dermal fibroblast spheroids Human Dermal Fibroblast spheroids	(Boutin and Hoffman-Kim, 2015; Boutin et al., 2018a) Kabadi et al., 2015 Costa et al., 2018b Costa et al., 2018a
	Sca*l*e Sca*l*eS Sca*l*eSQ	Urea Glycerol Triton X-100 Urea Sorbitol Glycerol DMSO Triton X-100	Neural cell spheres Various human cell spheroids Cancer cell spheroids patient-derived lung tumor organoid Breast cancer spheroids	Boutin and Hoffman-Kim, 2015 Nürnberg et al., 2020 Boutin et al., 2018b Takahashi et al., 2019 Grist et al., 2016
	SeeDB	D(-)-fructose	Breast cancer spheroids	Grist et al., 2016
	FUnGI	D(-)-fructose Glycerol Urea	Human colonic organoids	van Ineveld et al., 2020
	FRUIT	D(-)-fructose Urea	iPSC-derived human cerebral organoids co-cultured with Patient-derived glioblastoma cells	Krieger et al., 2020
	Fructose-glycerol (FG)	D-(-)-Fructose Glycerol	Human colonic organoids	Dekkers et al., 2019
	Sca*l*eCUBIC-1/2 (1^st^ gen. CUBIC) CUBIC-L/R (2^nd^ gen. CUBIC)	(Delipidation) Quadrol Urea Triton X-100 (RI matching) Triethanolamine Urea Sucrose (Delipidation) N-butyldiethanolamine Triton X-100 (RI matching) Nicotinamide N-methylnicotinamide Antipyrine	Tumor cell spheroids Cancer cell spheroids Human iPSC-derived ureteric bud organoids Matrigel-embedded tumor cell spheroids Human iPSC-derived cortico-striatal assembroids	Masson et al., 2015 Kang et al., 2020 Mae et al., 2020 Molley et al., 2020 Miura et al., 2020
	RTF	Triethanolamine Formamide	Brain organoids	Rakotoson et al., 2019
	FocusClear^TM*^	Diatrizoic acid Tween 20	Human intestinal crypt organoids	Chen et al., 2013
	PROTOS**	Diatrizoic acid N-methyl-D-glucamine Iodixanol	Murine intestinal organoids	Serra et al., 2019
	RapiClear^®^ ***		Murine intestinal organoids Pancreatic tumor cell spheroids Human iPSC-derived brain spheroids	Lallemant et al., 2020 Steinberg et al., 2020 Govindan et al., 2021
Hydrogel-tissue chemistry	CLARITY/PACT	Hydrogel embedding (Delipidation) SDS (RI matching) Histodenz^TM^ Glycerol	Tumor cell spheroids Murine/human cell spheroids Adipose-derived stem cell spheroids Human ESC-derived cerebral organoids Human iPSC-derived retinal organoids	Masson et al., 2015 Chen et al., 2016 Silva Santisteban et al., 2017 Sakaguchi et al., 2019 Cora et al., 2019
	SWITCH	Glutaraldehyde cross-linking (Delipidation) SDS (RI matching) Diatrizoic acid N-methyl-D-glucamine Iodixanol	Human ESC-derived cerebral organoids	Renner et al., 2017
	SHIELD	Polyepoxy cross-linking (Delipidation) SDS (RI matching) Diatrizoic acid N-methyl-D-glucamine Iodixanol	Human iPSC-derived cerebral organoids	Albanese et al., 2020
	ExM	Hydrogel embedding (RI matching) Expansion in water	Tumor cell spheroids	Edwards et al., 2020

*A commercialized proprietary reagent provided by CelExplorer Labs Co. (Hsinchu, Taiwan). **This reagent was initially developed for samples prepared by hydrogel-tissue chemistry. ***A commercialized proprietary reagent provided by SunJin Lab Co. (Hsinchu, Taiwan).

A side-by-side comparison of protocols would be helpful to identify the clearing method that is most appropriate for a particular application. Boutin and Hoffman-Kim compared early phase hydrophilic clearing protocols (*Clear^*T2*^*, Sca*l*eA2, and SeeDB) and concluded that *Clear*^*T2*^ is the most effective for clearing neural spheres (Boutin and Hoffman-Kim, 2015). However, in a later study, the authors also applied an updated Sca*l*e protocol (Sca*l*eS) for clearing of cancer cell spheroids (Boutin et al., 2018b). Cheung and colleagues compared SeeDB, *Clear*^*T2*^, and Sca*l*eSQ for adaptation to an on-chip spheroid processing system and concluded that SeeDB and Sca*l*eSQ were more effective to clear a sample than *Clear*^*T2*^, although there were some drawbacks of increased autofluorescence and sample expansion (Grist et al., 2016). Schöler, Bruder and colleagues compared the performance of the organic solvent reagent BABB with several other types of clearing reagents and found that BABB-based clearing proved to be both the fastest and most efficient for clearing of human midbrain organoids (Renner et al., 2020). Garfa-Traoré and colleagues compared several clearing methods (TDE, CUBIC, and RapiClear^®^) for murine intestinal organoids and obtained the best clearing and staining results with RapiClear^®^ (Lallemant et al., 2020). Rudolf and colleagues tested several hydrophilic reagents and mounting media (glycerol, Sca*l*eS, *Clear*^*T2*^, and CytoVista) for clearing of various spheroids and found that Sca*l*eS and a high concentration of glycerol (88% *RI* = 1.459) provided the best clearing results, while preserving the fluorescent signals and maintaining sample integrity, although various factors (i.e., size, complexity, and composition) affected the clearing results (Nürnberg et al., 2020). Another study suggested similar performance of glycerol (>85%) and RapiClear^®^ for clearing of pancreatic tumor spheroids (Steinberg et al., 2020). Lorenzo and colleagues demonstrated the effectiveness of both CUBIC and CLARITY for clearing of tumor cell spheroids (Masson et al., 2015).

These mixed results indicate that there is no “gold-standard” protocol for clearing of all cell reaggregates. As with the clearing of large tissue samples, it is necessary to choose an appropriate protocol in consideration of the pros and cons. The complexity of a reaggregate can affect the efficiency of optical clearing (Nürnberg et al., 2020) and thus may occasionally require delipidation. For example, Paşca and colleagues clearly and quantitatively reproduced cortico-striatal projections in human induced PSC (iPSC)-derived cortico-striatal assembloids with the use of the latest CUBIC-L/R procedure (Miura et al., 2020). Chung and colleagues applied SHIELD technology to single-cell and cytoarchitecture combined with multiple labeling methods for analysis of organoids (Albanese et al., 2020). ExM (Chen et al., 2015) enables super-resolution imaging together with improved clearing and staining results. Brismar and colleagues applied ExM to probe labeling and for high-resolution imaging of tumor cell spheroids, and found that as compared to simpler clearing protocols, ExM improved antibody penetration and image resolution in deeper regions (Edwards et al., 2020).

**FIGURE 1:**
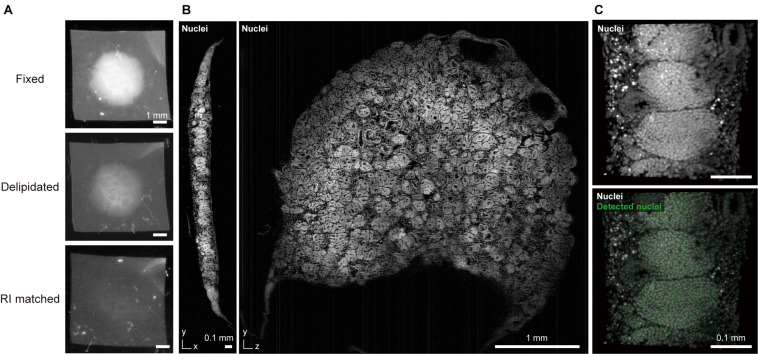
CUBIC-clearing and 3D imaging of a whole human kidney organoid. **(A)** A human iPSC-derived large kidney organoid (φ∼ 4 mm, thickness > 300 μm) (Takasato et al., 2016) is delipidated and RI-matched with a modified CUBIC-L/R protocol. **(B)** The cleared and propidium iodide-stained kidney organoid was imaged with a custom-built macrozoom LSFM (GEMINI system, Susaki et al., 2020) with a voxel resolution of ∼2.5 μm^3^. **(C)** A part of the organoid image was subjected to nuclear coordinate detection (3D Maxima analysis, modified from our previously reported method (Susaki et al., 2020), which can be used for further 3D analysis of the cell architecture.

Tailoring of key parameters (e.g., compound type, concentration, immersion time, and temperature) in the original protocol should also be taken into account. Correia and colleagues found that the molecular weight of polyethylene glycol in *Clear*^*T2*^ reagent can affect clearing and imaging quality (Costa et al., 2018b). Molley et al. (2020) used the Sca*l*eCUBIC-2 protocol for clearing of Matrigel-embedded microtumors with some modifications to timing, washing, and handling. Dekkers et al. (2019) designed a fructose-glycerol immersion method as a simple, non-toxic, optical clearing step for complete 3D imaging of fragile organoids. Later, the authors cleared human colonic organoids with their FUnGI clearing reagent that was originally developed for clearing of human cancer specimens (Rios et al., 2019; van Ineveld et al., 2020). Our group modified the delipidation stringency of our CUBIC protocol for clearing of large kidney organoids (Figure 1).

Other than clearing performance, the compatibility of the cell-labeling/probing methods and microscopy setup should also be considered, as discussed in the following sections.

### Labeling of Spheroids and Organoids With Fluorescent Proteins and Probes

Since tissue clearing alone cannot label objects in a 3D structure, appropriate cell/structure labeling with a fluorescent protein (FP) or histological staining is also required for observation. Therefore, it is crucial to consider the compatibility of the tissue clearing technique with various labeling methods.

Conventional organic solvents show weak retention of FP signals. When using reagents in this category with FP labeling, more FP-compatible protocols (Schwarz et al., 2015; Pan et al., 2016; Qi et al., 2019) should be considered. Tanaka and colleagues recently improved the ECi method for clearing of FP-labeled organoids (Masselink et al., 2019). Alternatively, clearing with hydrophilic reagents or tissue-hydrogel chemistry methods that generally retain FP signals should be considered. In this regard, some methods strictly compared the performance of keeping FP protein signals with other clearing protocols. For example, Ce3D has a superior ability to FP preservation when compared to major clearing methodologies (BABB, DISCO, Sca*l*e, Sca*l*eCUBIC, SeeDB, *Clear*^*T*^, CLARITY, PACT, and SWITCH) (Li et al., 2017).

Whole-mount labeling of organoids with molecular probes (antibodies and small chemical dyes) has also been widely applied in many organoid studies. However, probe diffusion in 3D specimens is generally problematic, even in “small” cell reaggregates. For example, Smyrek and Stelzer investigated multiple whole-mount spheroid immunostaining parameters, including permeabilization, incubation time, and temperature, based on classical immunostaining procedures and proposed parameter guidelines (Smyrek and Stelzer, 2017). However, the signal intensity and homogeneity were varied and dependent on the antibody type and treatment protocol. Schöler, Bruder and colleagues very recently reported staining, clearing, and quantification analysis of relatively large (> 800 μm diameter) organoids (Renner et al., 2020). While the procedure was successfully automated, the protocol required a long incubation time (total 12 days for primary and secondary antibody staining) with multiple renewals of the staining reagents.

Various tissue clearing methods for large-scale 3D staining have been developed with improved efficiency and homogeneity. Recent developments of improved and more versatile 3D staining protocols, such as iDISCO, AbSca*l*e/ChemSca*l*e, SWITCH, and CUBIC-HistoVIsion (Renier et al., 2014, 2016; Hama et al., 2015; Murray et al., 2015; Susaki et al., 2020), have enabled large-scale 3D tissue staining. iDISCO, with optimized permeabilization and staining steps, enables immunostaining of whole mouse embryos and brains. AbSca*l*e/ChemSca*l*e utilizes urea to facilitate probe penetration via Sca*l*e clearing technology. SWITCH modulates the kinetics of probe binding to tissue by two procedures: SWITCH-off (inhibits probe binding) and SWITCH-on (facilitates probe binding). In our latest study on CUBIC-HistoVIsion, biological tissue was modeled as an electrolyte gel for screening of multiple essential 3D staining conditions that provides highly optimized 3D staining of cleared specimens of an entire organ and the whole body. However, the current protocol is not applicable to multiplex immunolabeling. Alternative protocols (e.g., Ce3D, SWITCH) can be considered to avoid this drawback. The incorporation of these recent 3D staining strategies can overcome the drawbacks of labeling entire organoids.

Several studies have applied these recent 3D staining protocols for labeling of whole organoids. For example, Takagi and colleagues applied AbSca*l*e immunostaining together with Sca*l*eS clearing to patient-derived tumor organoids for *in vitro* evaluation of molecularly targeted drugs (Takahashi et al., 2019). Another study employed AbSca*l*e and Sca*l*eS for staining and clearing of various human cell spheroids (Nürnberg et al., 2020). In addition, Ergun and colleagues applied an iDISCO-based whole-organoid procedure to stain vascularized tumors and neural organoids (Wörsdörfer et al., 2019). Moreover, for staining of human cerebral organoids, Chung and colleagues applied a modified eFLASH protocol that enables homogeneous staining of 8–10 whole organoids simultaneously in 1–2 days (Yun et al., 2019). The versatile staining ability of their SCOUT method further supports system-level analysis of the framework of 3D organoids (Albanese et al., 2020).

As in the case of FP, it should be noted that retention of the stained signals is dependent on the clearing reagents, as some may remove a portion of the staining target, while others may alter the binding affinity or antigenicity (Lallemant et al., 2020; Matryba et al., 2020). For example, the intensive comparison by Li et al. (2017) showed that the preservation degree of antigenicity toward major cell type markers in the lymph node is varied among tested methods. Antigen retrieval methods have been incorporated in some 3D staining and clearing protocols (Messal et al., 2021). After staining, cross-linking is occasionally required to preserve binding of the probe when clearing a specimen (Susaki et al., 2020).

### 3D Imaging of Cleared Spheroids and Organoids

The acquisition of whole-organoid information requires a proper 3D imaging setup. Besides conventional line-scan imaging, such as confocal and two-photon microscopy, light-sheet fluorescence microscopy (LSFM) is especially useful for 3D observation of cleared whole specimens. LSFM excites the fluorescent signals within the cleared sample with a sheet-shaped illumination and acquires section images with a vertically positioned complementary metal-oxide semiconductor (cMOS) or charge-coupled device (CCD) camera. This setup enables collection of high-throughput 3D images of the entire sample with minimal photodamage. LSFM has thus been proposed as the optimal modality for high-speed, high-quality 3D imaging of cleared samples, including spheroids and organoids (Dodt et al., 2007; Pampaloni et al., 2007; Keller and Stelzer, 2008; Santi, 2011; Keller and Dodt, 2012; Costa et al., 2019). As compared with confocal and two-photon microscopy, experimental studies have reported the superior speed and depth of LSFM for imaging of 3D organoids (Lallemant et al., 2020).

Custom-built LSFM systems have been reported in earlier studies. For example, a series of studies reported organoid/spheroid clearing with LSFM imaging using a monolithic digital scanned laser light-sheet-based fluorescence microscope (mDSLM) (Keller et al., 2008; Schmitz et al., 2017; Smyrek and Stelzer, 2017) and a millimeter-scaled optofluidic lab-on-a-chip device, which integrates light-sheet illumination and a microfluidic channel, for imaging of cell spheroids (Paiè et al., 2016). Moreover, Lorenzo and colleagues improved the resolution of 3D spheroid images using a light-sheet setup with adaptive optics (Masson et al., 2015). A specific light-sheet setup, such as a dual-view inverted selective plane illumination microscope (Kumar et al., 2014; Eismann et al., 2020), open-top LSFM (Glaser et al., 2019), and single-objective LSFM (Li et al., 2014; Bouchard et al., 2015), have the potential to enable large-scale imaging for screening purposes owing to the compatibility of these systems for imaging of multiple organoids cultured in multi-well plates.

With the commercialization of LSFMs, the opportunities of 3D organoid imaging with clearing are beginning to expand. The ZEISS Lightsheet Z.1 has been one of the most popular commercial LSFMs so far, enabling high-throughput multi-view 3D imaging with an easy-to-use operation (Cora et al., 2019; Dekkers et al., 2019; Sakaguchi et al., 2019; Lallemant et al., 2020; Molley et al., 2020; Preusser et al., 2020). However, the RI range of the equipped objective lens is up to 1.48, which is incompatible with some transparent reagents with an RI of > 1.5 and thus can cause the formation of spherical aberrations when imaging large samples at higher magnifications. The recently released Lightsheet 7 improves this issue with an RI range of 1.33–1.58, allowing the imaging of samples up to 2 cm in size with the use of almost any clearing reagent.

Regardless of the type of microscope used, the design of the entire experimental workflow should be optimized by selecting an appropriate clearing method, microscope setup, objective lens specification, and image resolution. Compatibility of some organic solvents (e.g., dibenzyl ether in iDISCO) with a commercialized LSFM should be taken into account, due to their corrosive effects such as dissolution of glues used in the construction of objective lenses (McKey et al., 2020). A proper optical resolution should also be set to meet the experimental and analytical objectives while avoiding oversampling. A high numerical aperture (NA) objective lens with a short working distance can also interfere with volume imaging. RI discrepancies between objective lens coverage and clearing reagents can reduce imaging quality due to spherical aberrations.

Whole imaging of a large kidney organoid that was generated from human iPS cells is depicted in Figure 1 (Takasato et al., 2016). The sample was cleared with CUBIC reagent (final *RI* = 1.52) and 3D data were collected at a voxel resolution of 2.5 μm^3^ to detect cell coordinates for subsequent cellular architecture analysis. Low magnification macro-objectives (NA ∼ 0.1) provide reasonable image quality and data size (15.5 GB for raw 16-bit TIFF data, 1.6 GB for processed 8-bit TIFF data) for this purpose. Oversampling microscopic images with excessive magnification and higher NA objectives will produce redundant data sets. For example, if an image is captured at 2.5 times the voxel resolution, the data size will be an order of magnitude larger (2.5^3^ = 15.625), thereby burdening data storage and subsequent image analysis.

## Discussion: Prospects of Organoid Research With Tissue Clearing Technology

Any organoid application requires sensitive and accurate phenotyping. Here, organoid models of human diseases are discussed. For example, regarding the use of organoids to recapitulate microcephaly caused by the Zika virus (ZIKV), forebrain organoid size and neuronal proliferation have been assessed (Dang et al., 2016; Qian et al., 2016). That platform also showed an upregulation of the innate immune receptor Toll-like receptor 3 (TLR3) after ZIKV infection of human organoids. A pharmacological administration with a TLR3 competitive inhibitor reduced the phenotypic effects of ZIKV infection (Dang et al., 2016). A model of polycystic kidney disease was also replicated *in vitro* with the use of kidney organoids, where multiple cysts actually formed within the organoids generated from patient-derived iPSCs (Cruz et al., 2017; Low et al., 2019; Shimizu et al., 2020). Several drugs, such as thapsigargin, a non-competitive inhibitor of the sarco/endoplasmic reticulum Ca^2+^ ATPase, and CFTRinh–172, a selective CFTR channel inhibitor, are reported to inhibit the number and size of cysts in the disease model.

A recent study proposed that 3D information relevant to the pathophysiological processes and responses to perturbations (e.g., drug administration) is needed for further quantitative and comprehensive analysis of these *in vitro* disease models. Chung and colleagues developed a prominent SCOUT framework for whole 3D organoid phenotyping by clearing- and LSFM imaging-based multiple feature acquisition and atlas-independent analysis (Albanese et al., 2020). The use of SCOUT successfully extracted multiple features from the 3D dataset relevant to the developmental stages of the organoid and differences in protocols. Finally, SCOUT was applied to the ZIKV infection model to quantify the multiscale impact of ZIKV infection on brain development in the 3D datasets. The analysis produced a comprehensive quantification of the pathology, including cell loss, reduction of ventricles, and overall change in tissue reorganization. Assessment of other complex information, such as the 3D neural network structure, will potentially be integrated into future studies of cerebral organoids or cortico-striatal assembroids (Renner et al., 2017; Miura et al., 2020).

**FIGURE 2:**
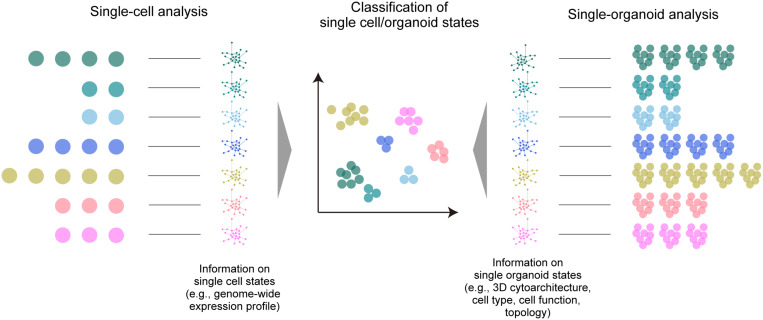
A large-scale multi-organoid analysis. As in the case of single-cell analysis, in which various omics data (e.g., a genome-wide expression profile) are collected to determine the cell type or state, the states of multiple single organoids can be analyzed by collecting data of the 3D cytoarchitecture, distributions of identified cell types, or topological features. This information can be finally used for the classification and clustering of the organoid population.

Apart from these disease models, imaging-based classification and clustering analysis of multiple organoid phenotypes for large-scale screening has also been proposed (Pampaloni et al., 2013) with a final aim to achieve classification and clustering of organoids based on the phenotype (i.e., organoid states), as with other omics analyses (Figure 2). Most studies so far have relied on the collection of various biological data within the organoids. As a representative approach, high-content imaging allows for high-throughput and multi-channel imaging data collection and analysis (Wenzel et al., 2014; Schmuck et al., 2017; Czerniecki et al., 2018; Durens et al., 2020). Recently, Lukonin et al. (2020) devised an imaging-based drug screening assay of approximately 450,000 intestinal organoids by extracting several features from the image (e.g., signal intensities of marker proteins, area, and circularity of the reaggregates) and clustering the organoids into 15 groups related to seven major phenotypes affected by the screened drugs. Tissue clearing techniques are beginning to be incorporated into such large-scale automated procedures for organoid screening (Silva Santisteban et al., 2017; Boutin et al., 2018b; Grenier et al., 2020; Renner et al., 2020; Rybin et al., 2021), which have been useful for the collection of comprehensive information across organoids and to improve screening accuracy. Histo-cytometry or proteomic imaging by multiplex and multi-modal labeling together with clearing can scale the amount of information (Murray et al., 2015; Li et al., 2017; Park et al., 2018).

The considerable data size and calculation throughput for clustering organoid phenotypes are possible drawbacks to large-scale screening with multi-modal information. Instead, organoid 3D data with less modality (e.g., a single channel of nuclear staining) may also provide sensitive information about the internal state. A simple topological analysis of all cell distribution within the 3D organoid by graph representation is a promising approach (Poli et al., 2019). A pioneering study by Stelzer and colleagues demonstrated multiscale analysis of individual and neighboring cells to the global topologies of optically cleared spheroids, and employed an analytical scheme inspired by graph theory and computational topology, in which all cell nuclei are segmented and represented as a cell graph for feature extraction (e.g., the relative position of each cell nucleus, the number of neighboring cells, and the distances to neighboring cells) (Schmitz et al., 2017). They also presented the possibility of multi-organoid clustering analysis based on the identified features. This proposed analytical scheme can alternatively offer an opportunity to classify the structural phenotypes (structural states) of multiple organoids based on mathematical topology analysis or machine learning-based feature extraction, which are occasionally independent of biological meanings. This scheme can, for example, enhance multi-organoid drug screening, which is currently dependent on classical dose-response curves (Broutier et al., 2017; Yan et al., 2018; Kopper et al., 2019; Takahashi et al., 2019). This 3D architecture-based classification and clustering methodology can also be readily combined with biological analyses of marker gene expression profiles, omics approaches, or physiological readout, further facilitating the extraction of essential molecular mechanisms. Tissue clearing technologies can fully support such large-scale, high-throughput topological analysis of multiple organoids in the future.

## Conclusion

The examples described here provide a clear perspective of tissue clearing techniques as excellent tools for organoid research to facilitate the collection of biological profiles using organoids, understanding of pathophysiological processes, and the development of new therapeutic tools. 3D spatial interrogation of organoids can also be applied in larger projects, such as the Organoid Cell Atlas of the Human Cell Atlas Project by providing references and workflows for comparing molecular expression patterns between organoids and cell populations in actual tissues/organs (Bock et al., 2021). Further accumulation of technical tips and applications will be needed in future efforts.

## Data Availability Statement

The raw data supporting the conclusions of this article will be made available by the authors, without undue reservation.

## Author Contributions

MT generated the kidney organoid. ES cleared and imaged the sample. Both authors contributed to the conception, writing, and review of the manuscript.

## Conflict of Interest

RIKEN and CUBICStars Co. have filed patents regarding this work, in which ES. was a co-inventor. ES was also a senior researcher employed by CUBICStars Co. The remaining author declares that the research was conducted in the absence of any commercial or financial relationships that could be construed as a potential conflict of interest.

## References

[B1] Albanese A. Swaney J. M. Yun D. H. Evans N. B. Antonucci J. M. Velasco S. (2020). Multiscale 3D phenotyping of human cerebral organoids. *Sci. Rep.* 10:21487. 10.1038/s41598-020-78130-7 33293587 PMC7723053

[B2] Aoyagi Y. Kawakami R. Osanai H. Hibi T. Nemoto T. (2015). A rapid optical clearing protocol using 2,2’-thiodiethanol for microscopic observation of fixed mouse brain. *PLoS One* 10:e0116280. 10.1371/journal.pone.0116280 25633541 PMC4310605

[B3] Bakutkin V. V. Maksimova I. L. Semyonova T. N. Tuchin V. V. Kon I. L. (1995). “Controlling optical properties of sclera,” in *Proceedings of the Ophthalmic Technologies V*, Vol. 2393 San Jose, CA. 10.1117/12.209841

[B4] Becker K. Jährling N. Saghafi S. Weiler R. Dodt H. U. (2012). Chemical clearing and dehydration of GFP expressing mouse brains. *PLoS One* 7:e33916. 10.1371/annotation/17e5ee57-fd17-40d7-a52c-fb6f86980def PMC3316521 22479475

[B5] Bock C. Boutros M. Camp J. G. Clarke L. Clevers H. Knoblich J. A. (2021). The Organoid Cell Atlas. *Nat. Biotechnol.* 39 13–17. 10.1038/s41587-020-00762-x 33384458 PMC7801253

[B6] Bouchard M. B. Voleti V. Mendes C. S. Lacefield C. Grueber W. B. Mann R. S. (2015). Swept confocally-aligned planar excitation (SCAPE) microscopy for high speed volumetric imaging of behaving organisms. *Nat. Photonics* 9 113–119. 10.1038/nphoton.2014.323 25663846 PMC4317333

[B7] Boutin M. E. Hoffman-Kim D. (2015). Application and assessment of optical clearing methods for imaging of tissue-engineered neural stem cell spheres. *Tissue Eng. Part C Methods* 21 292–302. 10.1089/ten.tec.2014.0296 25128373 PMC4346659

[B8] Boutin M. E. Kramer L. L. Livi L. L. Brown T. Moore C. Hoffman-Kim D. (2018a). A three-dimensional neural spheroid model for capillary-like network formation. *J. Neurosci. Methods* 299 55–63. 10.1016/j.jneumeth.2017.01.014 28143748

[B9] Boutin M. E. Voss T. C. Titus S. A. Cruz-Gutierrez K. Michael S. Ferrer M. (2018b). A high-throughput imaging and nuclear segmentation analysis protocol for cleared 3D culture models. *Sci. Rep.* 8:11135. 10.1038/s41598-018-29169-0 30042482 PMC6057966

[B10] Broutier L. Mastrogiovanni G. Verstegen M. M. A. (2017). Human primary liver cancer–derived organoid cultures for disease modeling and drug screening. *Nat. Med.* 23 1424–1435. 10.1038/nm.4438 29131160 PMC5722201

[B11] Chen F. Tillberg P. W. Boyden E. S. (2015). Expansion microscopy. *Science* 347 543–548. 10.1126/science.1260088 25592419 PMC4312537

[B12] Chen Y. Y. Silva P. N. Syed A. M. Sindhwani S. Rocheleau J. V. Chan W. C. W. (2016). Clarifying intact 3D tissues on a microfluidic chip for high-throughput structural analysis. *Proc. Natl. Acad. Sci. U.S.A.* 113 14915–14920. 10.1073/pnas.1609569114 27956625 PMC5206515

[B13] Chen Y. Tsai Y.-H. Liu Y.-A. Lee S.-H. Tseng S.-H. Tang S.-C. (2013). Application of three-dimensional imaging to the intestinal crypt organoids and biopsied intestinal tissues. *Sci. World J.* 2013 624342. 10.1155/2013/624342 24348177 PMC3848346

[B14] Chiang A. Liu Y. Chiu S. Hu S. Huang C. Hsieh C. (2001). Three−dimensional mapping of brain neuropils in the cockroach, *Diploptera punctata*. *J. Comp. Neurol.* 440 1–11. 10.1002/cne.1365 11745603

[B15] Cora V. Haderspeck J. Antkowiak L. Mattheus U. Neckel P. H. Mack A. F. (2019). A cleared view on retinal organoids. *Cells* 8:391. 10.3390/cells8050391 31035373 PMC6562974

[B16] Costa E. C. Moreira A. F. de Melo-Diogo D. Correia I. J. (2018a). ClearT immersion optical clearing method for intact 3D spheroids imaging through confocal laser scanning microscopy. *Opt. Laser Technol.* 106 94–99. 10.1016/j.optlastec.2018.04.002 29752799

[B17] Costa E. C. Moreira A. F. de Melo-Diogo D. Correia I. J. (2018b). Polyethylene glycol molecular weight influences the Clear^*T2*^ optical clearing method for spheroids imaging by confocal laser scanning microscopy. *J Biomed. Opt.* 23:055003. 10.1117/1.JBO.23.5.055003 29752799

[B18] Costa E. C. Silva D. N. Moreira A. F. Correia I. J. (2019). Optical clearing methods: an overview of the techniques used for the imaging of 3D spheroids. *Biotechnol. Bioeng.* 116 2742–2763. 10.1002/bit.27105 31282993

[B19] Cruz N. M. Song X. Czerniecki S. M. Gulieva R. E. Churchill A. J. Kim Y. K. (2017). Organoid cystogenesis reveals a critical role of microenvironment in human polycystic kidney disease. *Nat. Mater.* 16 1112–1119. 10.1038/nmat4994 28967916 PMC5936694

[B20] Czerniecki S. M. Cruz N. M. Harder J. L. Menon R. Annis J. Otto E. A. (2018). High-throughput screening enhances kidney organoid differentiation from human pluripotent stem cells and enables automated multidimensional phenotyping. *Cell Stem Cell* 22 929–940. 10.1016/j.stem.2018.04.022 29779890 PMC5984728

[B21] Dang J. Tiwari S. K. Lichinchi G. Qin Y. Patil V. S. Eroshkin A. M. (2016). Zika virus depletes neural progenitors in human cerebral organoids through activation of the innate immune receptor TLR3. *Cell Stem Cell* 19 258–265. 10.1016/j.stem.2016.04.014 27162029 PMC5116380

[B22] Davis R. P. Ng E. S. Costa M. Mossman A. K. Sourris K. Elefanty A. G. (2008). Targeting a GFP reporter gene to the MIXL1 locus of human embryonic stem cells identifies human primitive streak-like cells and enables isolation of primitive hematopoietic precursors. *Blood* 111 1876–1884. 10.1182/blood-2007-06-093609 18032708

[B23] Dekkers J. F. Alieva M. Wellens L. M. Ariese H. C. R. Jamieson P. R. Vonk A. M. (2019). High-resolution 3D imaging of fixed and cleared organoids. *Nat. Protoc.* 14 1756–1771. 10.1038/s41596-019-0160-8 31053799

[B24] Dent J. A. Polson A. G. Klymkowsky M. W. (1989). A whole-mount immunocytochemical analysis of the expression of the intermediate filament protein vimentin in Xenopus. *Development* 105 61–74. 10.1242/dev.105.1.61 2806118

[B25] Desmaison A. Guillaume L. Triclin S. Weiss P. Ducommun B. Lobjois V. (2018). Impact of physical confinement on nuclei geometry and cell division dynamics in 3D spheroids. *Sci. Rep.* 8:8785. 10.1038/s41598-018-27060-6 29884887 PMC5993719

[B26] Dodt H. U. Leischner U. Schierloh A. Jährling N. Mauch C. P. Deininger K. (2007). Ultramicroscopy: three-dimensional visualization of neuronal networks in the whole mouse brain. *Nat. Methods* 4 331–336. 10.1038/nmeth1036 17384643

[B27] Durens M. Nestor J. Williams M. Herold K. Niescier R. F. Lunden J. W. (2020). High-throughput screening of human induced pluripotent stem cell-derived brain organoids. *J. Neurosci. Methods* 335:108627. 10.1016/j.jneumeth.2020.108627 32032714

[B28] Edwards S. J. Carannante V. Kuhnigk K. Ring H. Tararuk T. Hallböök F. (2020). High-resolution imaging of tumor spheroids and organoids enabled by expansion microscopy. *Front. Mol. Biosci.* 7:1405. 10.3389/fmolb.2020.00208 33195398 PMC7543521

[B29] Eiraku M. Takata N. Ishibashi H. Kawada M. Sakakura E. Okuda S. (2011). Self-organizing optic-cup morphogenesis in three-dimensional culture. *Nature* 472 51–56. 10.1038/nature09941 21475194

[B30] Eismann B. Krieger T. G. Beneke J. Bulkescher R. Adam L. Erfle H. (2020). Automated 3D light-sheet screening with high spatiotemporal resolution reveals mitotic phenotypes. *J. Cell Sci.* 133:jcs245043. 10.1242/jcs.245043 32295847 PMC7286290

[B31] Ertürk A. Becker K. Jährling N. Mauch C. P. Hojer C. D. Egen J. G. (2012). Three-dimensional imaging of solvent-cleared organs using 3DISCO. *Nat. Protoc.* 7 1983–1995. 10.1038/nprot.2012.119 23060243

[B32] Foglietta F. Canaparo R. Muccioli G. Terreno E. Serpe L. (2020). Methodological aspects and pharmacological applications of three-dimensional cancer cell cultures and organoids. *Life Sci.* 254:117784. 10.1016/j.lfs.2020.117784 32416169

[B33] Garita-Hernandez M. Guibbal L. Toualbi L. Routet F. Chaffiol A. Winckler C. (2018). Optogenetic light sensors in human retinal organoids. *Front. Neurosci.* 12:789. 10.3389/fnins.2018.00789 30450028 PMC6224345

[B34] Glaser A. K. Reder N. P. Chen Y. Yin C. Wei L. Kang S. (2019). Multi-immersion open-top light-sheet microscope for high-throughput imaging of cleared tissues. *Nat. Commun.* 10:2781. 10.1038/s41467-019-10534-0 31273194 PMC6609674

[B35] Goranci-Buzhala G. Mariappan A. Gabriel E. Ramani A. Ricci-Vitiani L. Buccarelli M. (2020). Rapid and efficient invasion assay of glioblastoma in human brain organoids. *Cell Rep.* 31:107738. 10.1016/j.celrep.2020.107738 32521263

[B36] Govindan S. Batti L. Osterop S. F. Stoppini L. Roux A. (2021). Mass generation, neuron labeling, and 3D imaging of minibrains. *Front. Bioeng. Biotechnol.* 8:1436. 10.3389/fbioe.2020.582650 33598450 PMC7883898

[B37] Gradinaru V. Treweek J. Overton K. Deisseroth K. (2018). Hydrogel-tissue chemistry: Principles and applications. *Annu. Rev. Biophys.* 47 355–376. 10.1146/annurev-biophys-070317-032905 29792820 PMC6359929

[B38] Grenier K. Kao J. Diamandis P. (2020). Three-dimensional modeling of human neurodegeneration: brain organoids coming of age. *Mol. Psychiatry* 25 254–274. 10.1038/s41380-019-0500-7 31444473

[B39] Grist S. M. Nasseri S. S. Poon T. Roskelley C. Cheung K. C. (2016). On-chip clearing of arrays of 3-D cell cultures and micro-tissues. *Biomicrofluidics* 10:044107. 10.1063/1.4959031 PMC4958101 27493703

[B40] Hama H. Hioki H. Namiki K. Hoshida T. Kurokawa H. Ishidate F. (2015). ScaleS: an optical clearing palette for biological imaging. *Nat. Neurosci.* 18 1518–1529. 10.1038/nn.4107 26368944

[B41] Hama H. Kurokawa H. Kawano H. Ando R. Shimogori T. Noda H. (2011). Scale: a chemical approach for fluorescence imaging and reconstruction of transparent mouse brain. *Nat. Neurosci.* 14 1481–1488. 10.1038/nn.2928 21878933

[B42] Hou B. Zhang D. Zhao S. Wei M. Yang Z. Wang S. (2015). Scalable and DiI-compatible optical clearance of the mammalian brain. *Front. Neuroanat.* 9:19. 10.3389/fnana.2015.00019 25759641 PMC4338786

[B43] Kabadi P. K. Vantangoli M. M. Rodd A. L. Leary E. Madnick S. J. Morgan J. R. (2015). Into the depths: techniques for in vitro three-dimensional microtissue visualization. *Biotechniques* 59 279–286. 10.2144/000114353 26554505 PMC4804457

[B44] Kang W. Ferruzzi J. Spatarelu C.-P. Han Y. L. Sharma Y. Koehler S. A. (2020). Tumor invasion as non-equilibrium phase separation. *bioRxiv* [Preprint]. 10.1101/2020.04.28.066845 PMC8564056 34755092

[B45] Ke M.-T. Fujimoto S. Imai T. (2013). SeeDB: a simple and morphology-preserving optical clearing agent for neuronal circuit reconstruction. *Nat. Neurosci.* 16 1154–1161. 10.1038/nn.3447 23792946

[B46] Ke M.-T. Nakai Y. Fujimoto S. Takayama R. Yoshida S. Kitajima T. S. (2016). Super-resolution mapping of neuronal circuitry with an index-optimized clearing agent. *Cell Rep.* 14 2718–2732. 10.1016/j.celrep.2016.02.057 26972009

[B47] Keller P. J. Dodt H. U. (2012). Light sheet microscopy of living or cleared specimens. *Curr. Opin. Neurobiol.* 22 138–143. 10.1016/j.conb.2011.08.003 21925871

[B48] Keller P. J. Stelzer E. H. K. (2008). Quantitative in vivo imaging of entire embryos with Digital scanned laser light sheet fluorescence microscopy. *Curr. Opin. Neurobiol.* 18 624–632. 10.1016/j.conb.2009.03.008 19375303

[B49] Keller P. J. Schmidt A. D. Wittbrodt J. Stelzer E. H. K. (2008). Reconstruction of zebrafish early embryonic development by scanned light sheet microscopy. *Science* 322 1065–1069. 10.1126/science.1162493 18845710

[B50] Kim J. H. Jang M. J. Choi J. Lee E. Song K.-D. Cho J. (2018). Optimizing tissue-clearing conditions based on analysis of the critical factors affecting tissue-clearing procedures. *Sci. Rep.* 8:12815. 10.1038/s41598-018-31153-7 30143733 PMC6109102

[B51] Klingberg A. Hasenberg A. Ludwig-Portugall I. Medyukhina A. Männ L. Brenzel A. (2017). Fully automated evaluation of total glomerular number and capillary tuft size in nephritic kidneys using lightsheet microscopy. *J. Am. Soc. Nephrol.* 28 452–459. 10.1681/ASN.2016020232 27487796 PMC5280021

[B52] Kopper O. de Witte C. J. Lõhmussaar K. Valle-Inclan J. E. Hami N. Kester L. (2019). An organoid platform for ovarian cancer captures intra- and interpatient heterogeneity. *Nat. Med.* 25 838–849. 10.1038/s41591-019-0422-6 31011202

[B53] Krieger T. G. Tirier S. M. Park J. Jechow K. Eisemann T. Peterziel H. (2020). Modeling glioblastoma invasion using human brain organoids and single-cell transcriptomics. *Neuro. Oncol.* 22 1138–1149. 10.1093/neuonc/noaa091 32297954 PMC7594554

[B54] Ku T. Swaney J. Park J. Y. Albanese A. Murray E. Cho J. H. (2016). Multiplexed and scalable super-resolution imaging of three-dimensional protein localization in size-adjustable tissues. *Nat. Biotechnol.* 34 973–981. 10.1038/nbt.3641 27454740 PMC5070610

[B55] Kumar A. Wu Y. Christensen R. Chandris P. Gandler W. McCreedy E. (2014). Dual-view plane illumination microscopy for rapid and spatially isotropic imaging. *Nat. Protoc.* 9 2555–2573. 10.1038/nprot.2014.172 25299154 PMC4386612

[B56] Kuwajima T. Sitko A. A. Bhansali P. Jurgens C. Guido W. Mason C. (2013). ClearT: a detergent- and solvent-free clearing method for neuronal and non-neuronal tissue. *Development* 140 1364–1368. 10.1242/dev.091844 23444362 PMC3912244

[B57] Lallemant L. Lebreton C. Garfa-Traoré M. (2020). Comparison of different clearing and acquisition methods for 3D imaging of murine intestinal organoids. *J. Biol. Methods* 7:e141. 10.14440/jbm.2020.334 33564693 PMC7865078

[B58] Lancaster M. A. Renner M. Martin C.-A. Wenzel D. Bicknell L. S. Hurles M. E. (2013). Cerebral organoids model human brain development and microcephaly. *Nature* 501 373–379. 10.1038/nature12517 23995685 PMC3817409

[B59] Li T. Ota S. Kim J. Wong Z. J. Wang Y. Yin X. (2014). Axial plane optical microscopy. *Sci. Rep.* 4 7253. 10.1038/srep07253 25434770 PMC4248283

[B60] Li W. Germain R. N. Gerner M. Y. (2017). Multiplex, quantitative cellular analysis in large tissue volumes with clearing-enhanced 3D microscopy (Ce3D). *Proc. Natl. Acad. Sci. U.S.A.* 114 E7321–E7330. 10.1073/pnas.1708981114 28808033 PMC5584454

[B61] Li Y. Tang P. Cai S. Peng J. Hua G. (2020). Organoid based personalized medicine: from bench to bedside. *Cell Regen* 9:21. 10.1186/s13619-020-00059-z PMC7603915 33135109

[B62] Low J. H. Li P. Chew E. G. Y. Zhou B. Suzuki K. Zhang T. (2019). Generation of human PSC-derived kidney organoids with patterned nephron segments and a de novo vascular network. *Cell Stem Cell* 25 373–387. 10.1016/j.stem.2019.06.009 31303547 PMC6731150

[B63] Lukonin I. Serra D. Challet M. L. Volkmann K. Baaten J. Zhao R. (2020). Phenotypic landscape of intestinal organoid regeneration. *Nature* 586 275–280. 10.1038/s41586-020-2776-9 33029001 PMC7116869

[B64] Lundvall V. H. (1905). “Veiteres iiber demonstration embryonaler skelette,” in *Anatomischer Anzeiger*, ed. von Bardeleben K. (Jena: Verlag von Gustav Fischer).

[B65] Mae S.-I. Ryosaka M. Sakamoto S. Matsuse K. Nozaki A. Igami M. (2020). Expansion of human iPSC-derived ureteric bud organoids with repeated branching potential. *Cell Rep.* 32:107963. 10.1016/j.celrep.2020.107963 32726627

[B66] Masselink W. Reumann D. Murawala P. Pasierbek P. Taniguchi Y. Bonnay F. (2019). Broad applicability of a streamlined ethyl cinnamate-based clearing procedure. *Development* 146:dev166884. 10.1242/dev.166884 30665888 PMC7115989

[B67] Masson A. Escande P. Frongia C. Clouvel G. Ducommun B. Lorenzo C. (2015). High-resolution in-depth imaging of optically cleared thick samples using an adaptive SPIM. *Sci. Rep.* 5:16898. 10.1038/srep16898 26576666 PMC4649629

[B68] Matryba P. Sosnowska A. Wolny A. Bozycki L. Greig A. Grzybowski J. (2020). Systematic evaluation of chemically distinct tissue optical clearing techniques in murine lymph nodes. *J. Immunol.* 204 1395–1407. 10.4049/jimmunol.1900847 31953352

[B69] McCracken K. W. Catá E. M. Crawford C. M. Sinagoga K. L. Schumacher M. Rockich B. E. (2014). Modelling human development and disease in pluripotent stem-cell-derived gastric organoids. *Nature* 516 400–404. 10.1038/nature13863 25363776 PMC4270898

[B70] McKey J. Cameron L. A. Lewis D. Batchvarov I. S. Capel B. (2020). Combined iDISCO and CUBIC tissue clearing and lightsheet microscopy for in toto analysis of the adult mouse ovary†. *Biol. Reprod.* 102 1080–1089. 10.1093/biolre/ioaa012 31965156 PMC7186783

[B71] Messal H. A. Almagro J. Zaw T. M. Tedeschi A. Ciccarelli A. Blackie L. (2021). Antigen retrieval and clearing for whole-organ immunofluorescence by FLASH. *Nat. Protoc.* 16 239–262. 10.1038/s41596-020-00414-z 33247285

[B72] Miura Y. Li M.-Y. Birey F. Ikeda K. Revah O. Thete M. V. (2020). Generation of human striatal organoids and cortico-striatal assembloids from human pluripotent stem cells. *Nat. Biotechnol.* 38 1421–1430. 10.1038/s41587-020-00763-w 33273741 PMC9042317

[B73] Molley T. G. Wang X. Hung T.-T. Jayathilaka P. B. Yang J.-L. Kilian K. A. (2020). Geometrically structured microtumors in 3D hydrogel matrices. *Adv Biosyst* 4:e2000056. 10.1002/adbi.202000056 32402124

[B74] Murakami T. C. Mano T. Saikawa S. Horiguchi S. A. Shigeta D. Baba K. (2018). A three-dimensional single-cell-resolution whole-brain atlas using CUBIC-X expansion microscopy and tissue clearing. *Nat. Neurosci.* 21 625–637. 10.1038/s41593-018-0109-1 29507408

[B75] Murray E. Cho J. H. Goodwin D. Ku T. Swaney J. Kim S. Y. (2015). Simple, scalable proteomic imaging for high-dimensional profiling of intact systems. *Cell* 163 1500–1514. 10.1016/j.cell.2015.11.025 26638076 PMC5275966

[B76] Nürnberg E. Vitacolonna M. Klicks J. von Molitor E. Cesetti T. Keller F. (2020). Routine optical clearing of 3D-cell cultures: simplicity forward. *Front. Mol. Biosci.* 7:20. 10.3389/fmolb.2020.00020 32154265 PMC7046628

[B77] Pagliuca F. W. Millman J. R. Gürtler M. Segel M. Van Dervort A. Ryu J. H. (2014). Generation of functional human pancreatic β cells in vitro. *Cell* 159 428–439. 10.1016/j.cell.2014.09.040 25303535 PMC4617632

[B78] Paiè P. Bragheri F. Bassi A. Osellame R. (2016). Selective plane illumination microscopy on a chip. *Lab. Chip.* 16 1556–1560. 10.1039/C6LC00084C 27030116

[B79] Pampaloni F. Ansari N. Stelzer E. H. K. (2013). High-resolution deep imaging of live cellular spheroids with light-sheet-based fluorescence microscopy. *Cell Tissue Res.* 352 161–177. 10.1007/s00441-013-1589-7 23443300

[B80] Pampaloni F. Reynaud E. G. Stelzer E. H. K. (2007). The third dimension bridges the gap between cell culture and live tissue. *Nat. Rev. Mol. Cell Biol.* 8 839–845. 10.1038/nrm2236 17684528

[B81] Pan C. Cai R. Quacquarelli F. P. Ghasemigharagoz A. Lourbopoulos A. Matryba P. (2016). Shrinkage-mediated imaging of entire organs and organisms using uDISCO. *Nat. Methods* 13 859–867. 10.1038/nmeth.3964 27548807

[B82] Park Y. G. Sohn C. H. Chen R. McCue M. Yun D. H. Drummond G. T. (2018). Protection of tissue physicochemical properties using polyfunctional crosslinkers. *Nat. Biotechnol.* 37 73–83. 10.1038/nbt.4281 30556815 PMC6579717

[B83] Poli D. Magliaro C. Ahluwalia A. (2019). Experimental and computational methods for the study of cerebral organoids: a review. *Front. Neurosci.* 13:162. 10.3389/fnins.2019.00162 30890910 PMC6411764

[B84] Preusser F. dos Santos N. Contzen J. Stachelscheid H. Costa ÉT. Mergenthaler P. (2020). FRC-QE: A robust and comparable 3D microscopy image quality metric for cleared organoids. *bioRxiv* [Preprint]. 10.1101/2020.09.10.291286 PMC8479654 33693580

[B85] Qi Y. Yu T. Xu J. Wan P. Ma Y. Zhu J. (2019). FDISCO: Advanced solvent-based clearing method for imaging whole organs. *Sci Adv* 5:eaau8355. 10.1126/sciadv.aau8355 30746463 PMC6357753

[B86] Qian X. Nguyen H. N. Song M. M. Hadiono C. Ogden S. C. Hammack C. (2016). Brain-Region-specific organoids using mini-bioreactors for modeling ZIKV exposure. *Cell* 165 1238–1254. 10.1016/j.cell.2016.04.032 27118425 PMC4900885

[B87] Rakotoson I. Delhomme B. Djian P. Deeg A. Brunstein M. Seebacher C. (2019). Fast 3-D imaging of brain organoids with a new single-objective planar-illumination two-photon microscope. *Front. Neuroanat.* 13:77. 10.3389/fnana.2019.00077 31481880 PMC6710410

[B88] Reichman S. Slembrouck A. Gagliardi G. Chaffiol A. Terray A. Nanteau C. (2017). Generation of storable retinal organoids and retinal pigmented epithelium from adherent human iPS Cells in xeno-free and feeder-free conditions. *Stem Cells* 35 1176–1188. 10.1002/stem.2586 28220575

[B89] Renier N. Adams E. L. Kirst C. Wu Z. Azevedo R. Kohl J. (2016). Mapping of brain activity by automated volume analysis of immediate early genes. *Cell* 165 1789–1802. 10.1016/j.cell.2016.05.007 27238021 PMC4912438

[B90] Renier N. Wu Z. Simon D. J. Yang J. Ariel P. Tessier-Lavigne M. (2014). iDISCO: a simple, rapid method to immunolabel large tissue samples for volume imaging. *Cell* 159 896–910. 10.1016/j.cell.2014.10.010 25417164

[B91] Renner H. Grabos M. Becker K. J. Kagermeier T. E. Wu J. Otto M. (2020). A fully automated high-throughput workflow for 3D-based chemical screening in human midbrain organoids. *Elife* 9:e52904. 10.7554/eLife.52904.sa2 PMC7609049 33138918

[B92] Renner M. Lancaster M. A. Bian S. Choi H. Ku T. Peer A. (2017). Self−organized developmental patterning and differentiation in cerebral organoids. *EMBO J.* 17:e201694700. 10.15252/embj.201694700 28283582 PMC5430225

[B93] Rios A. C. Clevers H. (2018). Imaging organoids: a bright future ahead. *Nat. Methods* 15 24–26. 10.1038/nmeth.4537 29298292

[B94] Rios A. C. Capaldo B. D. Vaillant F. Pal B. van Ineveld R. Dawson C. A. (2019). Intraclonal plasticity in mammary tumors revealed through large-scale single-cell resolution 3D imaging. *Cancer Cell* 35:e6. 10.1016/j.ccell.2019.05.011 31185217

[B95] Rybin M. J. Ivan M. E. Ayad N. G. Zeier Z. (2021). Organoid models of glioblastoma and their role in drug discovery. *Front. Cell. Neurosci.* 15:4. 10.3389/fncel.2021.605255 33613198 PMC7892608

[B96] Sakaguchi H. Ozaki Y. Ashida T. Matsubara T. Oishi N. Kihara S. (2019). Self-organized synchronous calcium transients in a cultured human neural network derived from cerebral organoids. *Stem Cell Rep.* 13 458–473. 10.1016/j.stemcr.2019.05.029 31257131 PMC6739638

[B97] Santi P. A. (2011). Light sheet fluorescence microscopy: a review. *J. Histochem. Cytochem.* 59 129–138. 10.1369/0022155410394857 21339178 PMC3201139

[B98] Schmitz A. Fischer S. C. Mattheyer C. Pampaloni F. Stelzer E. H. (2017). Multiscale image analysis reveals structural heterogeneity of the cell microenvironment in homotypic spheroids. *Sci. Rep.* 7:43693. 10.1038/srep43693 28255161 PMC5334646

[B99] Schmuck M. R. Temme T. Dach K. de Boer D. Barenys M. Bendt F. (2017). Omnisphero: a high-content image analysis (HCA) approach for phenotypic developmental neurotoxicity (DNT) screenings of organoid neurosphere cultures in vitro. *Arch. Toxicol.* 91 2017–2028. 10.1007/s00204-016-1852-2 27722930

[B100] Schwarz M. K. Scherbarth A. Sprengel R. Engelhardt J. Theer P. Giese G. (2015). Fluorescent-protein stabilization and high-resolution imaging of cleared, intact mouse brains. *PLoS One* 10:e0124650. 10.1371/journal.pone.0124650 25993380 PMC4439039

[B101] Serra D. Mayr U. Boni A. Lukonin I. Rempfler M. Challet M. L. (2019). Self-organization and symmetry breaking in intestinal organoid development. *Nature* 569 66–72. 10.1038/s41586-019-1146-y 31019299 PMC6544541

[B102] Shimizu T. Mae S.-I. Araoka T. Okita K. Hotta A. Yamagata K. (2020). A novel ADPKD model using kidney organoids derived from disease-specific human iPSCs. *Biochem. Biophys. Res. Commun.* 529 1186–1194. 10.1016/j.bbrc.2020.06.141 32819584

[B103] Silva Santisteban T. Rabajania O. Kalinina I. Robinson S. Meier M. (2017). Rapid spheroid clearing on a microfluidic chip. *Lab Chip.* 18 153–161. 10.1039/C7LC01114H 29192297

[B104] Smyrek I. Stelzer E. H. K. (2017). Quantitative three-dimensional evaluation of immunofluorescence staining for large whole mount spheroids with light sheet microscopy. *Biomed. Opt. Exp.* 8 484–499. 10.1364/BOE.8.000484 28270962 PMC5330556

[B105] Spalteholz W. (1914). *Über das Durchsichtigmachen Von Menschlichen Und Tierischen Präparaten.* Leipzig: S. Hirzel.

[B106] Spence J. R. Mayhew C. N. Rankin S. A. Kuhar M. F. Vallance J. E. Tolle K. (2011). Directed differentiation of human pluripotent stem cells into intestinal tissue in vitro. *Nature* 470 105–109. 10.1038/nature09691 21151107 PMC3033971

[B107] Steinberg E. Orehov N. Tischenko K. Schwob O. Zamir G. Hubert A. (2020). Rapid clearing for high resolution 3D imaging of ex vivo pancreatic cancer spheroids. *Int. J. Mol. Sci.* 21 7703. 10.3390/ijms21207703 33081011 PMC7589457

[B108] Susaki E. A. Ueda H. R. (2016). Whole-body and whole-organ clearing and imaging techniques with single-cell resolution: toward organism-level systems biology in mammals. *Cell Chem. Biol.* 23 137–157. 10.1016/j.chembiol.2015.11.009 26933741

[B109] Susaki E. A. Shimizu C. Kuno A. Tainaka K. Li X. Nishi K. (2020). Versatile whole-organ/body staining and imaging based on electrolyte-gel properties of biological tissues. *Nat. Commun.* 11:1982. 10.1038/s41467-020-15906-5 32341345 PMC7184626

[B110] Susaki E. A. Tainaka K. Perrin D. Kishino F. Tawara T. Watanabe T. M. (2014). Whole-brain imaging with single-cell resolution using chemical cocktails and computational analysis. *Cell* 157 726–739. 10.1016/j.cell.2014.03.042 24746791

[B111] Tainaka K. Murakami T. C. Susaki E. A. Shimizu C. Saito R. Takahashi K. (2018). Chemical landscape for tissue clearing based on hydrophilic reagents. *Cell Rep.* 24 2196–2210. 10.1016/j.celrep.2018.07.056 30134179

[B112] Takahashi N. Hoshi H. Higa A. Hiyama G. Tamura H. Ogawa M. (2019). An in vitro system for evaluating molecular targeted drugs using lung patient-derived tumor organoids. *Cells* 8:481. 10.3390/cells8050481 31137590 PMC6562414

[B113] Takasato M. Er P. X. Becroft M. Vanslambrouck J. M. Stanley E. G. Elefanty A. G. (2014). Directing human embryonic stem cell differentiation towards a renal lineage generates a self-organizing kidney. *Nat. Cell Biol.* 16 118–126. 10.1038/ncb2894 24335651

[B114] Takasato M. Er P. X. Chiu H. S. Little M. H. (2016). Generation of kidney organoids from human pluripotent stem cells. *Nat. Protoc.* 11 1681–1692. 10.1038/nprot.2016.098 27560173 PMC5113819

[B115] Takasato M. Er P. X. Chiu H. S. Maier B. Baillie G. J. Ferguson C. (2015). Kidney organoids from human iPS cells contain multiple lineages and model human nephrogenesis. *Nature* 526 564–568. 10.1038/nature15695 26444236

[B116] Takebe T. Sekine K. Enomura M. Koike H. Kimura M. Ogaeri T. (2013). Vascularized and functional human liver from an iPSC-derived organ bud transplant. *Nature* 499 481–484. 10.1038/nature12271 23823721

[B117] Timmins N. E. Nielsen L. K. (2007). Generation of multicellular tumor spheroids by the hanging-drop method. *Methods Mol. Med.* 140 141–151. 10.1007/978-1-59745-443-8_8 18085207

[B118] Tuchin V. V. Maksimova I. L. Zimnyakov D. A. Kon I. L. Mavlyutov A. H. Mishin A. A. (1997). Light propagation in tissues with controlled optical properties. *J. Biomed. Opt.* 2 401–417. 10.1117/12.281502 23014964

[B119] Ueda H. R. Dodt H. U. Osten P. Economo M. N. Chandrashekar J. Keller P. J. (2020a). Whole-brain profiling of cells and circuits in mammals by tissue clearing and light-sheet microscopy. *Neuron* 106 369–387. 10.1016/j.neuron.2020.03.004 32380050 PMC7213014

[B120] Ueda H. R. Erturk A. Chung K. Gradinaru V. Chedotal A. Tomancak P. (2020b). Tissue clearing and its applications in neuroscience. *Nat. Rev. Neurosci.* 21 61–79. 10.1038/s41583-019-0250-1 31896771 PMC8121164

[B121] van Ineveld R. L. Ariese H. C. R. Wehrens E. J. Dekkers J. F. Rios A. C. (2020). Single-cell resolution three-dimensional imaging of intact organoids. *J. Vis. Exp.* 160:e60709. 10.3791/60709 32568249

[B122] Wang D. Haviland D. L. Burns A. R. Zsigmond E. Wetsel R. A. (2007). A pure population of lung alveolar epithelial type II cells derived from human embryonic stem cells. *Proc. Natl. Acad. Sci. U.S.A.* 104 4449–4454. 10.1073/pnas.0700052104 17360544 PMC1838621

[B123] Wei M. Shi L. Shen Y. Zhao Z. Guzman A. Kaufman L. J. (2019). Volumetric chemical imaging by clearing-enhanced stimulated Raman scattering microscopy. *Proc. Natl. Acad. Sci. U.S.A.* 116 6608–6617. 10.1073/pnas.1813044116 30872474 PMC6452712

[B124] Wenzel C. Riefke B. Grundemann S. Krebs A. Christian S. Prinz F. (2014). 3D high-content screening for the identification of compounds that target cells in dormant tumor spheroid regions. *Exp. Cell Res.* 323 131–143. 10.1016/j.yexcr.2014.01.017 24480576

[B125] Wörsdörfer P. Dalda N. Kern A. Krüger S. Wagner N. Kwok C. K. (2019). Generation of complex human organoid models including vascular networks by incorporation of mesodermal progenitor cells. *Sci. Rep.* 9:15663. 10.1038/s41598-019-52204-7 31666641 PMC6821804

[B126] Xia X. Zhang S.-C. (2009). Differentiation of neuroepithelia from human embryonic stem cells. *Methods Mol. Biol.* 549 51–58. 10.1007/978-1-60327-931-4_4 19378195 PMC2948207

[B127] Yan H. H. N. Siu H. C. Law S. Ho S. L. Yue S. S. K. Tsui W. Y. (2018). A Comprehensive human gastric cancer organoid biobank captures tumor subtype heterogeneity and enables therapeutic screening. *Cell Stem Cell* 23 882–897. 10.1016/j.stem.2018.09.016 30344100

[B128] Yang L. Soonpaa M. H. Adler E. D. Roepke T. K. Kattman S. J. Kennedy M. (2008). Human cardiovascular progenitor cells develop from a KDR+ embryonic-stem-cell-derived population. *Nature* 453 524–528. 10.1038/nature06894 18432194

[B129] Yu T. Zhu J. Li D. Zhu D. (2021). Physical and chemical mechanisms of tissue optical clearing. *iScience* 21:102178. 10.1016/j.isci.2021.102178 33718830 PMC7920833

[B130] Yu T. Zhu J. Li Y. Ma Y. Wang J. Cheng X. (2018). RTF: a rapid and versatile tissue optical clearing method. *Sci. Rep.* 8:1964. 10.1038/s41598-018-20306-3 29386656 PMC5792593

[B131] Yun D. H. Park Y.-G. Cho J. H. Kamentsky L. Evans N. B. Albanese A. (2019). Ultrafast immunostaining of organ-scale tissues for scalable proteomic phenotyping. *bioRxiv* [Preprint]. 10.1101/660373

[B132] Zimnyakov D. A. Tuchin V. V. Michin A. A. Kon I. L. Serov A. N. (1996). In-vitro human sclera structure analysis using tissue optical immersion effect. *Int. Soc. Opt. Photon. Ophthal. Technol. VI* 2673 233–243. 10.1117/12.240070

